# Opportunities for advancing science to inform tobacco regulation in an evolving tobacco landscape

**DOI:** 10.1038/s41380-024-02646-x

**Published:** 2024-07-02

**Authors:** Carlos Blanco, Benjamin Apelberg, Brian A. King, Wilson M. Compton

**Affiliations:** 1 National Institute on Drug Abuse, National Institutes of Health, Rockville, MD USA; 2 Center for Tobacco Products, Food and Drug Administration, Silver Springs, MD USA

**Keywords:** Psychiatric disorders, Prognostic markers

Cigarette smoking has been declining among U.S. adults for over a half century, and among U.S. youth since the late 1990s [1]. Yet, tobacco use remains the single largest cause of preventable disease and death in the US [2]. Each year, nearly 500,000 smoking-attributable deaths occur in the US, and the annual direct healthcare and lost productivity costs associated with smoking exceed $600 billion nationally [1, 3].

Despite significant reductions in self-reported cigarette smoking among US adults, disparities persist, including among individuals with psychiatric disorders [4]. For example, in 2019, current smoking was higher among those reporting a depressive episode (24.2% vs. 17.6%) or a substance use disorder (35.8 vs. 16.8%) compared to those not reporting these disorders [5]. Research indicates similar disparities among individuals with anxiety or personality disorders [5, 6].

The ultimate public health impact of tobacco products is a function of multiple factors, including initiation among non-users, cessation among current users, and relapse among former users. Further, while the overwhelming health burden of tobacco use is caused by combustible products, including cigarettes, the public health response is complicated by the continually evolving tobacco landscape. We outline four areas of opportunity in tobacco regulatory science – which is research to inform the regulation of tobacco products – to help clinicians understand existing and proposed regulations (e.g., under what conditions regulations are most impactful), educate patients, and advance efforts to reduce tobacco-related disease and death.

## Diverse landscape

Opportunities exist to better understand more recently introduced tobacco products, including e-cigarettes. Clinical trial data suggest that e-cigarettes can help some adults who smoke quit [7], but population data suggest their effect may vary by multiple factors, including product characteristics and motivation to stop smoking. Identifying the impact of such factors, particularly by population subgroups, is important. For example, understanding the characteristics of e-cigarettes that increase the likelihood of transitioning completely from cigarette smoking, and the lifetime course of e-cigarette use and its potential long-term effects (including respiratory function, cardiovascular outcomes, and cancer risk) are important clinical and research questions. Further, use of electronic devices for consumption of cannabis or other substances, and the design of optimal approaches to minimize youth initiation, are also of interest. Further research on these topics could help inform both clinical and public health practice.

There is also a need to better understand the influence of other novel products, such as heated tobacco products and nicotine pouches, and how changes in the marketplace, including marketing of new products and newer nicotine formulations, influence use, particularly among individuals who smoke. For example, nicotine concentration and formulation may be key factors that influence the ability of adults who smoke cigarettes to transition completely away from cigarettes [8]. Conversely, these factors could also influence the likelihood of addiction among non-users, particularly youth. As tobacco products continue to evolve, robust science on the use, addiction liability, and health impacts of different products is important to inform regulation and educate patients.

More refined knowledge about the adverse effects of tobacco use, particularly across populations groups, could help inform approaches targeted toward high-risk groups, including in clinical practice. For example, identification of characteristics, such as flavors, that increase the appeal or addiction liability of certain tobacco products can help inform regulation and inform patients. Similarly, identification of characteristics that contribute to facilitating cessation or discourage initiation would also be informative.

Opportunities also exist to better understand biomarkers of different tobacco products and their role as indicators of exposure, measures of disease severity, or as a way to identify disease mechanisms. Observational and experimental studies could benefit from enhanced collection of biospecimens to facilitate the integration of biological and clinical data.

### Disparities

Longstanding tobacco-related disparities exist based on racial or ethnic group status, socioeconomic status, sexual orientation and gender identity, geography, behavioral health status, and other factors. Therefore, continued surveillance of disparities, as well as assessments of their drivers and evaluations of tailored interventions, is essential. Addressing the effects of tobacco-related disparities – related to both product use and policy implementation and enforcement – is particularly critical.

Tobacco regulatory science can provide evidence to address inequities. For example, regulatory science was key to informing the product standards proposed by FDA in to prohibit menthol in cigarettes and all characterizing flavors in cigars. These proposed rules could save hundreds of thousands of lives over time, with an especially pronounced benefit for populations with higher prevalence of use, including certain racial and ethnic populations, low-income populations, and LGBTQ+ individuals. FDA has also announced its intent to cap nicotine levels in cigarettes and certain other combustible tobacco products with the goal of reducing youth use, addition, and death, which can further reduce disparities [9]. Such a policy has also been considered elsewhere, including impact on disparities; a modeling study in New Zealand found that reduced nicotine in cigarettes could be effective in decreasing smoking prevalence and eliminating disparities [10]. Additionally, experiencing discrimination is related to tobacco use across the lifespan, and environmental inequities can contribute to increased risk of tobacco use and adverse consequences. Research approaches and regulations that help eliminate these inequities, in addition to increased knowledge about the effects of regulations to inform clinicians and patients, are essential. For example, given the higher prevalence of smoking among individuals with psychiatric disorders, regulations that target products with particularly high addictive liability could be particularly beneficial among this population.

### Policy evaluation

The tobacco product and regulatory landscapes at the international, national, state and local levels are dynamic and require ongoing evaluation. Such evaluation allows for documentation of intended and unintended impacts, as well as barriers and facilitators to policy implementation. The evaluation of international, state, and local policies can provide valuable evidence to inform the likely impacts of Federal regulatory action, such as tobacco product standards. In addition to randomized designs, instrumental variable approaches, such as use of interstate variation in policy design or implementation, may provide information on the impact of those policies, and how to refine them. For example, randomized trials in clinical samples show that lowering nicotine content levels is associated with lower addictive potential in smoked tobacco products and subsequent changes in smoking behavior [11]. Prospective observational studies could help further clarify the impact of such a policy, particularly across broader population groups.

Implementation science offers opportunities to understand variations in the application of regulations, document the health consequences of those variations, and suggest refinements to the regulations. Implementation science can guide the adoption, scalability, and sustainability of preventive and treatment interventions for tobacco use and associated health consequences. For example, clinicians can identify barriers and facilitators to the implementation of policies as well as their impact on patients. This may include, for example, regulations on where tobacco use is allowed or providing incentives to prevent tobacco use or treat tobacco use disorder among individuals with or at risk of psychiatric disorders.

### Education and communication

Misinformation has undermined the credibility of evidence-based processes in several areas of science, including tobacco regulation. A coordinated effort will be important to restore public confidence and ensure access to truthful information about use of tobacco products and associated health consequences. Health communication and education efforts to prevent youth use of all tobacco product are particularly critical. For example, combating attractive depictions of tobacco product use in films and social media, and educating younger generations on the harmful effects of tobacco use remain essential. Consortia comprising patients, clinicians, researchers, regulators and public health officials could help identify the optimal information strategies, as is done in other areas of medicine. Clinicians, as trusted advisors to their patients, can play a unique role in these approaches.

Opportunities also exist to educate adult individuals who smoke about the continuum of risk of tobacco products, with combustible products having the greatest risk. For example, Black, Hispanic, and low-income individuals who smoke appear more likely to believe that e-cigarettes are more harmful than combustible cigarettes, and to have more positive tobacco-related social norms than those who are White and have higher-income [9]. It is essential that communication efforts are fully informed by science that documents the effectiveness of the messaging and does not have adverse consequences such as increased initiation of use among youth.

## Conclusion

Tobacco product regulation plays an essential role in reducing the adverse toll of tobacco use. Well-conducted scientific research is critical to inform regulatory policy development, as well as efforts by clinicians. Examining changes in the tobacco product landscape, working to achieve health equity, studying the effect of policy changes, and employing evidence-based education and communication strategies provide unique opportunities to advance population health, eliminate inequities, and protect the youth.
